# Immunomodulatory Effects of a Concoction of Natural Bioactive Compounds—Mechanistic Insights

**DOI:** 10.3390/biomedicines9111522

**Published:** 2021-10-22

**Authors:** Vani Gangwar, Amar Garg, Karan Lomore, Kalyani Korla, Shruthi S. Bhat, Raghavendra P. Rao, Mohamed Rafiq, Rajesh Kumawath, Babu V. Uddagiri, Venkatesh V. Kareenhalli

**Affiliations:** 1 MetFlux Research Private Limited, Mumbai 400078, India; vani.gangwar@metflux.in (V.G.); amar.kgarg166@gmail.com (A.G.); karan.lomore@metflux.in (K.L.); kalyani.korla@metflux.in (K.K.); 2 Himalaya Wellness Company, Bengaluru 562162, India; shruthi.bhat@himalayawellness.com (S.S.B.); raghavendra.pr@himalayawellness.com (R.P.R.); dr.rafiq@himalayawellness.com (M.R.); rajesh.kumawat@himalayawellness.com (R.K.); dr.babu@himalayawellness.com (B.V.U.); 3 Indian Institute of Technology Bombay (I.I.T.B.), Mumbai 400076, India

**Keywords:** Apigenin, Quercetin, Betulinic acid, Oleanolic acid, β-Sitosterol, inflammation, oxidative stress, cancer, autoimmune diseases, neutropenia

## Abstract

Natural bioactive compounds derived from plant-based products are known for their biological immunomodulatory activities. They possess systemic pleiotropic effects, minimal side effects, and very low toxicities. Plant-based bioactive compounds have tremendous potential as natural therapeutic entities against various disease conditions and act as anti-inflammatory, antioxidant, anti-mutagenic, anti-microbial, anti-viral, anti-tumour, anti-allergic, neuroprotective, and cardioprotective agents. A herbal formulation extract including five biologically active compounds: Apigenin, Quercetin, Betulinic acid, Oleanolic acid, and β-Sitosterol can impart several immunomodulatory effects. In this review, we systematically present the impact of these compounds on important molecular signaling pathways, including inflammation, immunity, redox metabolism, neuroinflammation, neutropenia, cell growth, apoptosis, and cell cycle. The review corroborates the beneficial effect of these compounds and shows considerable potential to be used as a safer, more cost-effective treatment for several diseases by affecting the major nodal points of various stimulatory pathways.

## 1. Introduction

Herbs have been a rich source of bioactive compounds for centuries to prevent and treat numerous diseases because of their therapeutic properties, multi-targeted efficacy, and low toxicity. In addition, their value as novel therapeutic agents has shown promise in contemporary research. Herbal medicines are characterized as one of the most important fields of medicines all over the world. Several plant family species such as Symplocaceae, Fabaceae, Liliaceae, Alangiaceae, Moraceae, Verbanaceae, and many others consist of bioactive compounds such as Apigenin, Quercetin, Betulinic acid, Oleanolic acid, and β-Sitosterol, which may have individual as well as synergistic immunomodulatory effects [1,2]. Many bioactive compounds and formulations have been studied for their efficacy and mechanism of action; however, their systemic effects and conjugated outcomes are yet to be deciphered.

Different groups of these natural bioactive compounds, such as flavonoids, triterpenoids, and phytosterols, exhibit immunomodulatory effects on the molecular pathways involved in the immune response against various disease conditions [3]. A herbal formulation extract containing key phytoconstituents such as (a) Apigenin (API), (b) Quercetin (QU), (c) Betulinic acid (BA), (d) Oleanolic acid (OA), and (e) β-Sitosterol (BS) (shown in Table 1) can be used as a concoction, as they have been widely studied for their immunomodulatory properties. Various drugs, such as Immusante, which is a herbal formulation containing extracts of *Symplocos racemosa* Roxb (Symplocaceae) and *Prosopis glandulosa* Torr (Fabaceae), have been reported to contain these bioactive compounds along with additional phytoconstituents in substantial amounts [4].

API and QU are flavonoids that demonstrate various biological properties such as antioxidant, anti-mutagenic, anti-inflammatory, anti-microbial, anti-viral, anti-tumour, anti-allergic, neuroprotective, and cardioprotective actions. They are, therefore, potential therapeutic entities [5,6,7,8,9,10]. Other functions include regulation of angiogenesis, wound healing, smooth muscle cell proliferation, fibrosis, and inflammation [11]. BA is a naturally occurring triterpenoid known for multiple bioactivities, such as anti-tumour, anti-HIV, and hepatoprotective activities [12]. BA is often used in phytopharmacology as a diuretic, antimycotic and anti-inflammatory remedy [13]. OA has important pharmacological properties, such as antioxidant, microbicide, antidiabetic, anti-inflammatory, hypolipidemic, and anti-atherosclerotic actions. They restrict the development of different types of cancer and neurodegenerative disorders [14]. BS exhibits a range of therapeutic effects such as antinociceptive, anxiolytic sedative effects, analgesic, immunomodulatory, anti-microbial, anti-cancer, anti-inflammatory, protective effects against nonalcoholic fatty liver disease (NAFLD), lipid-lowering effect, hepatoprotective, protective effect on respiratory diseases, wound healing effect, antioxidant and antidiabetic activity [15,16].

In the present review, we provide a detailed overview of the systemic effects of these phytochemicals on various signaling pathways relevant to immunomodulation and cell growth dynamics. In addition, holistic effects of the natural compounds on the pathogenesis network, including scenarios such as inflammation, oxidative stress, cancer microenvironment, autoimmune disorders, and other immune responses, are presented.

## 2. Inflammation and Immunity

Bioactive compounds are known to contribute significantly to the anti-inflammatory response. Different inflammatory pathways, such as the mitogen-activated protein kinase (MAPK), janus kinase/signal transducers and activators of transcription (JAK/STAT), and specifically the nuclear factor kappa-light-chain-enhancer of activated B cells (NF-κB) pathway, help regulate the inflammatory responses by generating inflammatory cytokines and mediators, cell proliferation, cell survival, T-cell differentiation, and dendritic cell (DC) maturation. Previous studies indicated a positive effect of all the selected phytochemicals in inflammatory pathways, discussed in later sections.

The activation of the inflammatory signaling pathways leads to the release of many pro-inflammatory mediators, such as tumour necrosis factors, interleukins, cyclooxygenases (COX) and anti-inflammatory interleukins such as IL-10. The role of these inflammatory mediators is to direct other immune cells at the site of infection, including circulating neutrophils that improve microbial killing through the production of interferons (IFN)-γ, proteases, and reactive oxygen species (ROS). Elimination of the foreign/endogenous agent and activation of the effector cells to dismiss the production of inflammatory mediators efficiently reduces inflammation and leads back to homeostasis. Continuous unresolved inflammation might lead to many chronic or degenerative diseases. Therefore, targeting underlying inflammation may be therapeutic in most degenerative disorders as many of them have minimal to no cure at this point [5].

**Figure 1:**
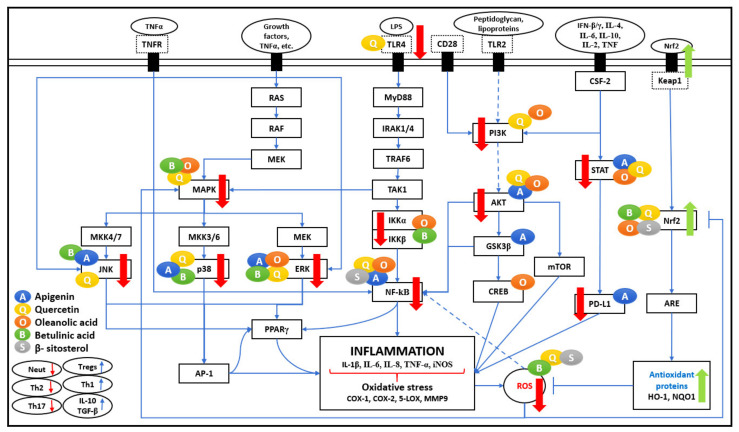
Inflammation pathogenesis network. The five bioactive compounds detailed in this review show a range of immunomodulatory activities. Various research articles have reported the multifaceted effects of bioactive compounds on significant pathways such as NF-κB, MAPK, PI3K/AKT, JAK-STAT, and the Nrf2 pathway. These bioactive compounds are shown as coloured circles and are indicated alongside their respective molecular targets (Blue: Apigenin, Yellow: Quercetin, Orange: Oleanolic acid, Green: Betulinic acid, Grey: β-Sitosterol). The bold red arrows indicate the downregulation of respective pathways, and bold green arrows indicate the upregulation of the antioxidant pathway. The entities of bold square boxes represent different signaling molecules/complexes. The entities of dotted square boxes represent different receptors for signaling pathways. The entities of circle boxes represent the inducers/activators of the particular pathways (top) and immune responses (bottom left). → represents direct activation, **--->** represents indirect activation, and **⊣** represents inhibition.

Figure 1 represents the effect of five bioactive compounds on major pathways producing pro-inflammatory cytokines and enzymes that contribute to increased immune response, leading to inflammation and oxidative stress.

### 2.1. NF-κB Pathway

The activation of NF-κB, a transcription factor, is mediated by Lipopolysaccharide (LPS) and pro-inflammatory cytokines such as TNF-α and IL-1. It leads to the relay activation of Myeloid differentiation primary response 88 (MyD88) and causing autophosphorylation of Interleukin-1 receptor-associated kinase (IRAK), which associate with tumour necrosis factor receptor-associated factor (TRAF6). As shown in Figure 1, all of these actions cause activation of Transforming growth factor β-activated kinase 1 (TAK1), which leads to phosphorylation of IκB kinase (IKK), an upstream regulator of NF-κB. The Toll-like receptor (TLR4) mediated MyD88 signaling pathway plays a major role in the relay activation of the NF-κB pathway. It is observed that QU negatively regulates LPS-induced TLR4 signaling. It reduces TLR4 expression and avoids NF-κB translocation to the nucleus in macrophages and human peripheral blood mononuclear cells (PBMCs), thus improving the anti-inflammatory response [20].

**Figure 2:**
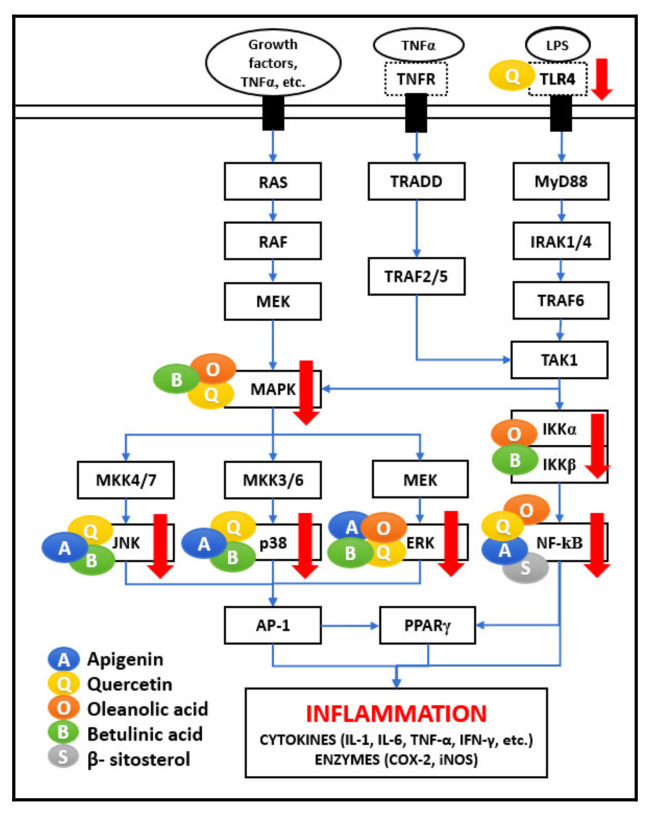
NF-κB and MAPK network. The five bioactive compounds detailed in this review show a range of immunomodulatory activities on these two pathways. The major effect of these compounds is seen for MAPK, JNK, p38, ERK, and NF-κB. The bioactive compounds are shown as coloured circles and are indicated alongside their respective molecular targets (Blue: Apigenin, Yellow: Quercetin, Orange: Oleanolic acid, Green: Betulinic acid, Grey: β-Sitosterol). The bold red arrows indicate the downregulation of respective pathways. The entities of bold square boxes represent different signaling molecules/complexes. The entities of dotted square boxes represent different receptors for signaling pathways. The entities of circle boxes represent the inducers/activators of the particular pathways. → represents direct activation.

As represented in Figure 1 and Figure 2, many different signaling molecules, such as TLR4, TNF, FasL, and TRAIL, cause IKK complex activation, resulting in IkBa phosphorylation and degradation by the proteasome [21]. Various studies reported that API, OA, BA, and BS administration inhibits NF-κB (IkBa) kinase (IKKß) and phosphorylation of IkBa, which leads to the suppression of NF-κB activation [14,20,22,23,24]. TNFR activation by TNF-α is also important for TAK1 activation, and consequently, activation of NF-κB signaling [6]. It is perceived that Th-1 derived cytokines such as IL-2, IFN-γ, and IL-12 improve cellular immunity, whereas Th-2 derived cytokines such as IL-4, IL-5, IL-6 employ negative immunoregulatory effects on cellular immunity. Therefore, the immuno-stimulatory beneficial impact in the presence of API, QU, and BA is mediated by inducing Th-1 derived cytokine, IFN-γ, and inhibiting Th-2 derived cytokine, IL-4, and LPS/TNFR induced TNF-α [7,9,13].

**Figure 3:**
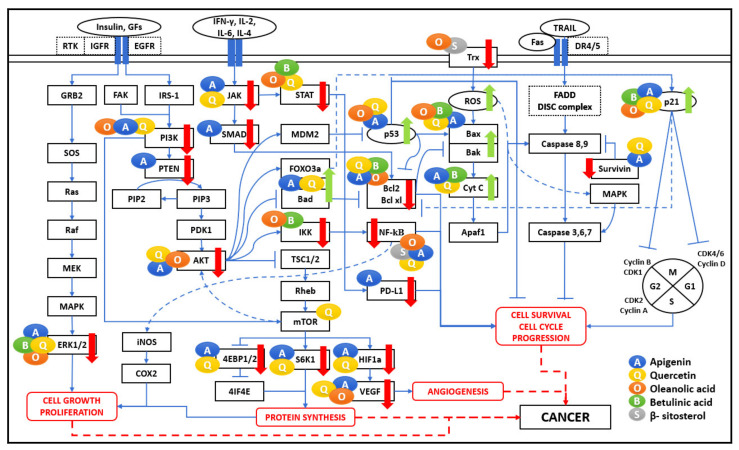
Cancer pathogenesis network. The five bioactive compounds detailed in this review show a range of immunomodulatory activities. Various research articles reported their scattered effects on significant pathways such as NF-κB, MAPK, PI3K/AKT/mTOR, JAK-STAT pathways regulating cell proliferation, angiogenesis, protein synthesis, cell survival, and cell cycle progression. These bioactive compounds are shown as coloured circles and are indicated alongside their respective molecular targets (Blue: Apigenin, Yellow: Quercetin, Orange: Oleanolic acid, Green: Betulinic acid, Grey: β-Sitosterol). The bold red arrows indicate the downregulation of respective pathways, and bold green arrows indicate the upregulation of the apoptosis pathway. The entities of bold square boxes represent different signaling molecules/complexes. The entities of dotted square boxes represent different receptors for signaling pathways. The entities of circle boxes represent the inducers/activators of the particular pathways. → represents direct activation, **--->** represents indirect activation, and ⊣ represents inhibition.

In the cancer microenvironment and oxidative stress, as shown in Figure 3, the NF-κB pathway prevents cell death via the activation of target genes, such as pro-survival genes (Bcl-2, Bcl-xL, survivin), cell cycle genes, vascular endothelial growth factor (VEGF), inflammatory cytokines, tumour metastasis genes, COX-2 and inducible NO synthase (iNOS) synthesis responses involving the nuclear factor erythroid-2-related factor 2 (Nrf2) pathway [7,22]. In various studies, QU, OA, and BA were found to significantly inhibit the production of NO, iNOS, IL-6, and the NF-κB in LPS-stimulated macrophages [13,25,26]. API inhibits several signaling pathways, including NF-κB and MAPK, representing anti-cancer effects [5]. The NF-κB pathway plays a significant role in cancer cell survival by letting tumour cells escape apoptosis as a downstream effect of Protein kinase B (PKB), also known as AKT pathway activation, leading to inflammation and oxidative stress, which causes cancer cell survival and proliferation through increased expression of cell cycle-related VEGF, inflammatory cytokines, and metastatic genes [21]. API has shown growth inhibitory properties in breast cancer via apoptosis promotion via blocking NF-κB signaling in breast cancer cells with HER2-overexpression [25].

Other immune responses include macrophages which are heterogeneous and plastic contributing towards inflammation; there are at least two significant macrophage populations: M1 cells are known to be pro-inflammatory and efficient producers of effector molecules (ROS and NO) and inflammatory cytokines (IL-1, TNF-α, IL-6), and M2 cells are anti-inflammatory with low IL-12, low IL-23, and high IL-10. M1 and M2 cells have diverse chemokine and chemokine receptor repertoires and thus orchestrate different immune responses. It is seen that QU and API may indirectly prevent inflammation by affecting peroxisome proliferator-activated receptor gamma (PPARγ) activity, antagonizing the NF-κB pathway or Activator protein 1 (AP-1) transcriptional activation of inflammatory genes, thereby reducing inflammation by successfully differentiating infiltrating macrophages to an anti-inflammatory M2 phenotype [6,9,27].

NF-κB is the central link in the regulation of inflammation at the cellular and molecular levels and has an essential role in regulating the synthesis of several key proteins involved in the activation and maintenance of the inflammatory state, maintaining the intricate balance of pro-inflammatory and anti-inflammatory pathways [28]. The myriad of effects exhibited by selected bioactive compounds may act synergistically to boost the protective effect of the immune system, thus contributing to enhanced therapeutic effects.

### 2.2. MAPK Pathway

MAPKs are protein-serine/threonine kinases, comprising c-Jun N-terminal kinases (JNKs), p38s, and extracellular Signal-Regulated Kinases (ERKs). MAPKs are controlled by various signals such as hormones, cytokines, growth factors, and endogenous stress, mediating various important cellular processes such as proliferation, differentiation, motility, stress response, apoptosis, and survival. As shown in Figure 2, MAPK activation is also mediated by TAK1 activation in response to LPS and TNF-α stimulus. Upon activation, JNKs phosphorylate several targets, generating the AP-1 transcription complex. AP-1 activation by various stimulatory mechanisms and activation of JNK, p38, and ERK pathway is responsible for producing inflammatory mediators such as TNF-α, interleukins (IL-6, IL-8, IL-1), matrix metalloproteinases (MMPs), and collagenase-1 causing inflammation. The JNK/AP-1 axis activation is involved in the pathogenesis and progression of several diseases, including cancer [21]. Activated p38 also contributes to AP-1 activation [6]. Studies have proven that QU and BA suppressed the activation of phosphorylated p38 MAP kinase [21,24].

Another mechanism includes activating the receptors by growth factors, such as epidermal growth factor (EGF). The binding of EGF leads to the activation of EGFR, which triggers the activation of Ras. Ras activates Raf, which phosphorylates MAPK/ERK kinase (MEK)1/2, later activating MAP kinases or MKK signaling. Studies showed that QU suppressed the activation of phosphorylated ERK kinase [20]. QU has a regulatory effect on immune cells functional properties, which the ERK-MAP kinase signal pathway may mediate in human mitogen-activated PBMC and purified T lymphocytes [15]. The Ras/Raf/MEK/MAPK pathway activation also triggers the AP-1 pathway [27]. AP-1 binds to AP-1 binding motifs in promoters in the DNA and increases the expression of pro-inflammatory factors, such as TNF-α, IL-1, IFN-γ, and MMPs. Compounds such as BA have protective effects by regulating c-Jun N-terminal kinase (JNK), p38, and ERK in MAPK signaling the transduction pathway [29]. QU inhibits MAPK/AP-1 inflammatory mediators. Treating human macrophages with QU reduces inflammation by reducing gene expression for TNF-α, IL-6, IL-8, IL-1, and by preventing levels of COX-2 and lipoxygenase (LOX) activities [23].

A combination of API, QU, OA, and BA can modulate NF-κB and MAPK pathways to reduce the inflammatory mediators. Since the mechanism of action is different in different cases, it is expected to have a strong, multifaceted effect on the immune system.

## 3. Oxidative Stress

Oxidative stress plays a vital role in the commencement of numerous human chronic diseases such as diabetes, atherosclerosis, cardiovascular diseases, myocardial infarction, rheumatoid arthritis, cancer, chronic inflammation, ageing, and several other neurodegenerative disorders. Furthermore, as shown in Figure 1 and Figure 3, ROS induces transcription factors such as NF-κB, MAPK, AP-1, p53, HIF-1α, PPARγ, STAT-3, and Nrf2 in cancer and inflammatory diseases.

### Nrf2 Pathway

The Nrf2 signaling pathway is a classic antioxidative stress-related pathway leading to upregulation of the antioxidant defence mechanisms. Under normal conditions, Nrf2 is kept in the cytoplasm by Keap1 in the form of Keap1-Nrf2 complexes. However, oxidative stress results in the decoupling of the Keap1-Nrf2 complex and released Nrf2 enters the nucleus and mediates the expression of antioxidant genes.

Multiple studies have demonstrated that QU, BA, and OA administration lead to upregulation of the Nrf2 signaling pathway, contributing to the increased HO-1 level inhibiting inflammatory mediators such as iNOS, IL-6, and IL-1ß [13,14,28,29,30,31,32,33]. Nrf2 controls antioxidative stress enzymes and drug-metabolizing enzymes (DMEs), such as glutathione S-transferase (GST) and NAD (P) H: quinone oxidoreductase 1 (NQO1). Studies have shown that QU and BA directly interact with Nrf2 as part of the NQO1 induction process, increasing Nrf2 protein levels [10,33]. Nrf2 activity is induced by binding to a motif termed antioxidant response element (ARE) in the promoter region of antioxidant enzymes encoding genes [31]. It is observed that API regulated the Nrf2/ARE pathway in cancer patients [6]. In cells challenged with oxidative stress by LPS treatment, QU treatment has increased p38 MAPK expression and inhibited NO production [31]. BA can improve oxidative stress by preventing the expression of p38, JNK, and ERK proteins, and therefore it can have protecting effects against oxidative damage [31,34]. Intracellular ROS was markedly decreased by pre-treatment with BA and BS and had a dose-dependent inhibitory effect [13,20].

Recent studies have defined the role of Nrf2 in cancer, obesity, metabolic syndrome, diabetic nephropathy, retinopathy, and neuropathy. In addition, the Nrf2 pathway plays regulatory roles in energy metabolism, mitochondrial function, and cellular redox balance. All five bioactive compounds API, QU, OA, BS, and BA act as antioxidants by impacting the Nrf2 pathway, and therefore a concoction of these phytochemicals can prove beneficial against diseases such as diabetes and cancer where oxidative stress increases complications.

## 4. Cancer Microenvironment

Tumour cells show numerous mechanisms to evade the immune system. The comprehensive interactions of the five bioactive compounds API, QU, OA, BS, and BA at several nodal points of various signaling pathways are shown in Figure 3. It lists all the major interactions captured in different types of cancer cell lines.

Beneficial effects of various phytochemicals altering the cancer microenvironment have been reported in different cell lines under various cancer conditions. These compounds inhibit cell cycle progression (via downregulation of RAS/RAF/MEK/ERK and PI3K/mTORC1 pathways); cell differentiation and angiogenesis (via downregulation of VEGF and HIF-1α pathway); and cell survival by modulating p53 expression, death receptor expression and altering the balance of pro-apoptotic (Bax) versus anti-apoptotic (Bcl-2) proteins. Interestingly, all the processes are unviolated for non-tumourigenic cells. In addition, these natural bioactive compounds also inhibit NF-κB, MAPK, JAK/STAT, and PI3K/AKT signaling, inhibiting inflammation and proliferation by downregulating pro-inflammatory mediators such as TNF-α, IL-6, COX-2, and PDL1. The broad-spectrum effects of these bioactive compounds can be seen through their interaction with several pathways, including cell cycle, apoptosis, growth factor, and ROS. It should be noted that these pathways are the target sites for several therapeutic drugs.

### 4.1. PI3K/PTEN/AKT/mTOR Signaling

The PI3K/PTEN/AKT/mTOR pathway can become unusually activated in several human cancers. Its activation can enhance cancer cell proliferation, tumour growth, angiogenesis, and survival, making it the most common route through which bioactive components induce apoptosis in cancer cells [35]. The signaling cascades get stimulated by either B and T cell receptors, receptor tyrosine kinases, cytokines, G-protein-coupled receptors (GPCRs), integrins, and other stimuli such as insulin and other growth factors. These stimuli act on their respective receptors to promote a relay of activation from the recruitment of IRS1 and the production of phosphatidylinositol (3,4,5)-trisphosphate (PIP3) through the activation of phosphoinositide 3-kinases (PI3K). PI3Ks catalyze the conversion of phosphatidylinositol (4,5)-bisphosphate (PIP2) to PIP3, and Phosphatase and tensin homolog (PTEN) converses its reaction. The PIP2 conversion recruits and activates the AKT, promoting mTOR activation [6]. PI3K, AKT, and mTOR are oncogenic proteins.

In contrast, PTEN acts as a tumour suppressor involved in cell-cycle regulation, preventing cells from growing and dividing too quickly. The loss or mutation of PTEN is associated with increased tumour development in many organs [27]. AKT is an oncogene highly expressed in human cancers and can be well-thought-out as the vital and convergent point of several growth signaling pathways. Phytochemicals such as API can block AKT function in different cancer cell types by reducing PI3K activity by obstructing the ATP-binding site of PI3K and then inhibiting AKT kinase activity [10,22]. API has shown growth inhibitory properties in breast cancer via apoptosis promotion by eliminating PI3K and AKT kinase activity [25]. QU treatment also decreased cell migration that was mediated by the suppression of AKT phosphorylation [27]. At the molecular level, QU induces apoptosis by triggering AKT-mTOR signaling interfering with protein synthesis [10,22].

Hypoxia-Inducible Factor (HIF-1α) is the transcriptional regulator of the adaptive response to hypoxia. Overexpression of HIF-1α seen in several cancers is responsible for drug resistance and higher mortality. HIF-1α promotes transcription of downstream genes essential for angiogenesis, such as VEGF, CREB-1, and also, for glycolysis, the GLUT-1 glucose transporter. API targets HIF-1α in several cancers such as ovarian, prostate, and lung cancer [25]. QU targets the VEGF signaling pathway, synergistically inhibiting cell invasion and proliferation in prostate cancer cell lines [33]. At the molecular level, QU-induced apoptosis can be modulated by HIF-1α signaling [16].

PI3K/AKT pathway is altered in many cancer types, causing enhanced activation of signaling cascades associated with excessive cellular growth, proliferation, and survival, acting on a varied range of downstream effectors, including murine double minutes 2 (Mdm2), FOXO, and SK-3,6 [31]. FOXO3a, a transcription factor and a tumour suppressor, is one of the PI3K/AKT signaling pathways downstream targets and is negatively controlled by AKT. Activation of FOXO3a is linked to poor diagnosis in a broad range of cancers. It is observed in various studies that API treatment induces FOXO3a expression by reducing AKT phosphorylation and then upregulating the expression of p21 and p27, FOXO3a target genes, which inhibits cancer cell proliferation [21].

The Ras/ERK signaling pathway is responsible for changes in cell morphology, differentiation, growth, proliferation, and neoplastic transformation. Many studies have shown that bioactive compounds can apply anti-angiogenic effects by regulating the expression of VEGF, MMPs, and EGFR and inhibiting the proangiogenic signaling pathways such as the ERK pathway. The anti-metastatic impact of novel synthetic OA derivatives leads to downregulation of the VEGF/JNK/ERK/NF-κB cascade and downregulated production and expression of inflammatory mediators such as TNF-α, IL-1ß, IL-6, and GM-CS [18]. API, BA, and QU inhibited highly invasive cells proliferation and acted as a therapeutic agent in cancer [24].

**Figure 4:**
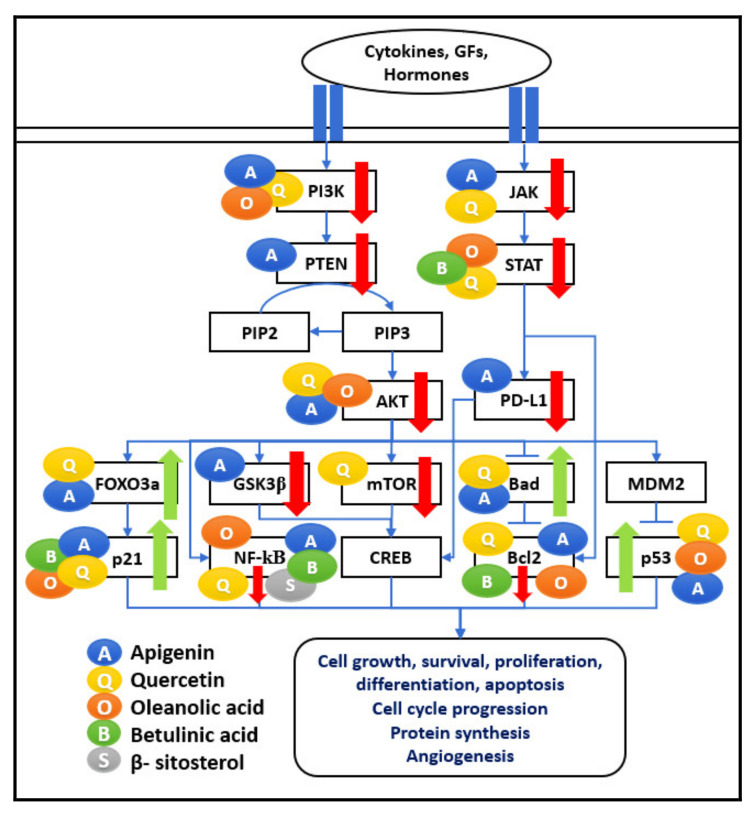
PI3K/AKT/mTOR and JAK/STAT network. The five bioactive compounds detailed in this review show a range of immunomodulatory activities. Various research articles have reported their scattered effects on significant pathways such as PI3K/AKT/mTOR, JAK-STAT pathways regulating cell proliferation, angiogenesis, protein synthesis, cell survival, and cell cycle progression. These bioactive compounds are shown as coloured circles and are indicated alongside their respective molecular targets (Blue: Apigenin, Yellow: Quercetin, Orange: Oleanolic acid, Green: Betulinic acid, Grey: β-Sitosterol). The bold red arrows indicate the downregulation of respective pathways, and bold green arrows indicate the upregulation of the particular pathway. The entities of bold square boxes represent different signaling molecules/complexes. The entities of dotted square boxes represent different receptors for signaling pathways. The entities of circle boxes represent the inducers/activators of the particular pathways. → represents direct activation and ⊣ represents inhibition.

A combination of API, QU, OA, and BA induces apoptosis in cancer cells by targeting the nodal points of the PI3K/AKT pathway. The nodal points considered in the network represent the major signaling mechanisms leading to cell survival gene expression with different approaches, e.g., Ras-Raf1/MEK/ERK, mTOR, and NF-κB leading to upregulation of inflammation, cell progression, protein synthesis, and angiogenesis, as shown in Figure 4. These phytochemicals regulate numerous cell functions, including cell apoptosis and survival, protein synthesis, cell growth, cell cycle, proliferation, and metabolism. Therefore, the anti-cancer properties of selected bioactive compounds can be considered for treatments to have an immunomodulatory effect on tumourigenic cells.

### 4.2. JAK-STAT Pathway

During the inflammation process, JAK-STAT signaling is activated by cytokines and coordinates the proliferation and differentiation of immune cells. Various STAT proteins have pro- or anti-inflammatory properties. The JAK1/STAT3 pathway plays a major role in inflammation, cell transformation, and carcinogenesis. The pro-inflammatory cytokines can increase STAT3 expression through a positive feedback loop. This cascade of events has multiple immune suppressive effects. Bioactive compounds such as API enhance the immune response by reducing JAK2 and STAT5 phosphorylation in cancer-associated cell lines. API also inhibits STAT3 and its direct target, VEGF, which has an immunosuppressive function [6]. They can also reduce tumour cell proliferation, sensitize the apoptotic pathways and reduce VEGF expression. API has shown growth inhibitory properties in breast cancer via apoptosis promotion via blocking STAT3 signaling in breast cancer cells with HER2-overexpression [25]. Another bioactive compound, BA, can reduce the activation of STAT3 and affect the STAT3/HIF-1α/VEGF signal pathway [22].

In a cancer environment, STAT3 can be a significant contributor to tumourigenic activity. This activity occurs due to the up-regulation of STAT3 in tumour cells, increasing the concentration of IL-6, IL-10, and IL-12. Literature studies have revealed the effect of QU blocking IL-12-induced phosphorylation of JAKs, TYKs, and STATs, resulting in reduced IL-12-induced T cell proliferation and Th1 differentiation [15]. The programmed cell death 1 (PD1) protein is generally expressed in immune cells, and its receptors PDL1 and PDL2 are widely expressed on the surface of dendritic cells or macrophages. PDL1 is highly expressed in many types of cancer cells and contributes to cancer cell immune evasion. Therefore, one strategy to stimulate immune surveillance against cancer cells is to target the expression of STAT-mediated activation of PD1/PDL1 in cancer cells. API, QU, and BA have been shown to inhibit STAT3 and IL-6 mediated signaling and expression of PDL1. API could target STAT1, resulting in the inhibition of IFN-γ-induced PDL1 expression [22,36,37,38].

Therefore, as shown in Figure 4, the JAK-STAT pathway is a major activator for producing inflammatory mediators, and oncogenic factors act as the target for the bioactive compounds such as API, QU, and BA. Therefore, combining such natural compounds can downregulate multiple outcomes of pro-inflammatory cytokines and carcinogens and thus function as anti-cancer agents.

### 4.3. Apoptosis Pathway

**Figure 5:**
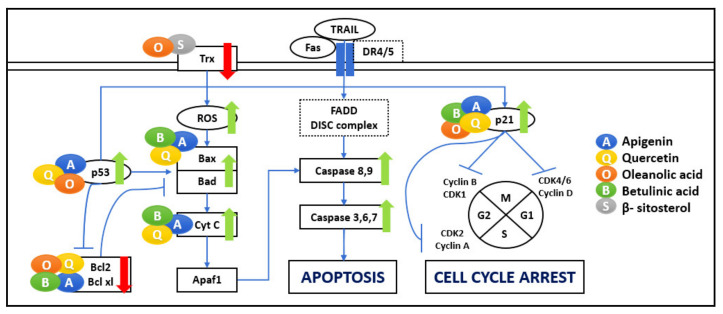
Apoptosis and cell cycle arrest. The five bioactive compounds show a range of immunomodulatory activities towards apoptosis and the cell cycle. Their effects on significant pathways include regulation of cell survival and cell cycle progression. These bioactive compounds are shown as coloured circles and are indicated alongside their respective molecular targets (Blue: Apigenin, Yellow: Quercetin, Orange: Oleanolic acid, Green: Betulinic acid, Grey: β-Sitosterol). The bold red arrows indicate the downregulation of respective pathways, and bold green arrows indicate the upregulation of the particular pathway. The entities of bold square boxes represent different signaling molecules/complexes. The entities of dotted square boxes represent different receptors for signaling pathways. The entities of circle boxes represent the inducers/activators of the particular pathways. → represents direct activation and ⊣ represents inhibition.

Apoptosis plays an essential role in maintaining tissue homeostasis and is highly conserved among diverse species. Malfunctioning apoptosis signaling might lead to various compulsive conditions such as human cancers. Cancer cells have a tendency to inactivate the mitochondrial intrinsic and extrinsic pathways of apoptosis [9]. In the cancer context, apoptosis is critical, and natural compounds regulation of pro-apoptotic and anti-apoptotic proteins is well studied (Figure 5).

Intrinsic apoptosis occurs inside the mitochondria, where its outer membrane is permeabilized and leads to Cytochrome c (Cyt c) release into the cytoplasm, facilitating apoptosome formation and activating caspase-dependent signaling pathways leading to apoptosis [12,19]. Various studies show that API’s effect has offered growth inhibitory properties in breast cancer by apoptosis promotion via activating the caspase cascade [25]. In addition to delaying cell growth by directly affecting important cell cycle modulators, QU can induce apoptosis via the mitochondrial pathway by triggering Cyt c in the cytoplasm [39,40]. Various studies were carried out to elucidate the mechanism of BA-mediated anti-tumour activity, including apoptosis by mitochondrial outer membrane permeabilization [9].

Extrinsic apoptosis, however, occurs outside the mitochondria and starts with the death receptors, which are cell membrane receptors known as Fas Receptor (FASR), Death Receptor 4/5 (DR4/5), and TNFR [6]. Death receptor activation causes the recruitment of adaptor FADD and activation of the initiator caspase-8, which broadcasts the death signal to caspase-3 [9]. API enhances the TRAIL-induced apoptosis by controlling DR4/DR5, AKT, ERK, and NF-κB signaling [8,22].

Molecularly, Bcl-2 families, regulating the balance of pro-apoptotic (Bax) and anti-apoptotic (Bcl-2) proteins, determine whether a cell survives or undergoes apoptosis [38]. Differences in the ratio of anti-apoptotic vs. pro-apoptotic Bcl-2 proteins may disturb the balance, favouring tumour cell survival instead of cell death. Bioactive compounds such as API block the nuclear translocation of NF-κB, thereby suppressing the expression of tumorigenic genes such as Bcl-2 and Bcl-xL [8,22]. Various studies were carried out to elucidate the mechanism of BA-mediated anti-tumour activity, including the regulation of induced apoptosis by Bcl-2 family proteins [9]. BA activates apoptosis by causing the upregulation of Bax, the downregulation of Bcl-2, and the activation of cleaved caspase-3, caspase-8, and caspase-9, and Cyt c [8]. OA upregulates the apoptotic genes Bax, caspase-9, and caspase-3, whereas the anti-apoptotic gene NF-κB–induced Bcl-2 are downregulated [18].

The Bcl-2 family is a p53 target. Bax, the pro-apoptotic protein, is upregulated in many systems during p53-mediated apoptosis. The p53 tumour suppressor was initially recognized as the “guardian of the genome” based on suppressor gene function’s ability to induce apoptosis. It is the main regulator of cell response to various stresses, including DNA damage and irregular growth regulation. p53 inactivation blocks apoptosis and facilitates the cell cycle, leading to uncontrolled proliferation and growth of cells. Activated p53 initiates a cascade of events that result in either growth arrest at one of the cell cycle checkpoints or apoptosis, leading to the elimination of genetically altered cells, therefore employing its tumour-suppressor function. Growth arrest is fundamentally provoked through the up-regulation of the genes that encode for inhibitors of cell-cycle progression, including p21 and p27 by p53. Bioactive compounds such as QU and API increase p53 expression, which strongly supports the evidence of the influence of flavonoids on cancer cell apoptosis, acting as isolated treatments or combined with other therapies [6]. API has shown growth inhibitory properties in breast cancer via apoptosis promotion via modulation of the p14ARF-Mdm2-p53 pathway [25]. In various cellular models, QU alleviates p53 both at mRNA and protein levels [36]. OA induce apoptosis by activation of the p53-induced caspase-mediated pro-apoptotic pathway. OA treatment can also upregulate p21 and p27 gene expression [18].

ROS-mediated apoptosis pathway plays an important role in the cell death process. ROS can lead to cell senescence or death at higher levels [8]. Cancer cells have a higher ROS occurrence than normal cells, which is a striking strategy to kill the cancer cells. Recently, stimulating ROS generation by compounds such as BA has been a productive strategy to target cancer cells [9]. The thioredoxin-thioredoxin reductase (Trx/TrxR) system has also been found to play a critical role in cancer. Enhanced expression of Trx1 and TrxR1 are reported in numerous cancer cells to control ROS homeostasis, promote cell growth and encourage apoptotic resistance. Trx/Trx1 reductase has given the impression of a new drug target modulating oxidative stress in cancer cells. OA can inhibit TrxR enzyme activity by regulating ROS levels, mediating ER stress and mitochondrial dysfunction, leading to cell cycle arrest and apoptosis [39]. BS constrains the protein expression of Trx/TrxR1, which triggers ROS accumulation in and activation of apoptotic cell death [11].

Overall, it is evident that the optimal functioning of the apoptosis pathway is an essential requirement to keep cancer metastasis in check and causing cell death in the required manner. The selected natural bioactive compounds, API, QU, OA, BS, and BA, act as anti-cancer agents by impacting the apoptosis pathway through different mechanisms. Therefore, a concoction of these elements can be beneficial against diseases such as cancer, where altered cell death increases complications.

### 4.4. Cell Cycle Arrest

As discussed in the previous section, a key feature of cancer is its uncontrolled and rapid cell division. As shown in Figure 5, the cell cycle has four programmed steps—named G1, S, G2, and M—in which the cell increases in size, duplicates its genetic material, prepares for division, and divides, respectively. One of the abnormalities in cancer cells is proliferation overriding the checkpoints of the cell cycle. Various studies report that cyclin-dependent kinases (CDKs) are significant regulators of cell cycle progression, immune cell activation, neo-angiogenesis in addition to inflammation. Numerous cancers are associated with activation of CDKs, because of mutation in CDK genes or CDK inhibitor genes. Therefore, targeting the growth of cancer cells is essential for suppressing cancer. Flavonoids such as API and QU have been shown to induce cell cycle arrest at checkpoints G1/S and G2/M through CDK inhibition in human cancer cells [5]. QU can control the cell cycle by directly binding various molecular targets and, depending on the cell type and tumour origin, it blocks the cell cycle at G2/M or the G1/S transition and by blocking the cell phase at the G2/M or G0/G1 checkpoint, blocks cell growth by inducing cell arrest at G2/M phase [22,28]. BS activates apoptosis in leukemic cancer cell lines by encouraging G2/M arrest [11].

Another important effect of bioactive compounds is to regulate the cell cycle by modulating several molecular targets, including p21, cyclin B, p27, CDKs, and topoisomerase II [36]. p21 is activated by p53 and promotes the inhibition of cyclin B, and E. p21 exerts inhibitory activity on several CDKs. p21 inhibits CDK2-cyclin E, thus inhibiting progression into and through the S phase. p21 also inhibits CDK2-cyclin A and CDK1-cyclin B, important for development through the S phase and G2. p27 employs several effects on the cell cycle and can also inhibit the complexes CDK4-cyclin D, and CDK6-cyclin D. OA treated cells also show a decrease in Cyclin D1 expression [18]. API and QU can regulate the cell cycle by suppressing cyclin B1 and its activating partners, Cdc2 and Cdc25c, and increasing cell cycle inhibitors, p53 and p21, cyclin A, cyclin B, and CDK1 [38,41].

## 5. Autoimmune Diseases/Neuroinflammation

**Figure 6:**
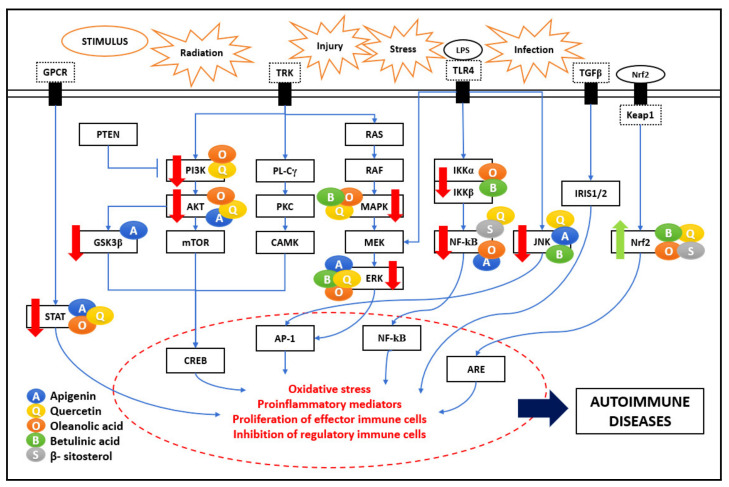
Autoimmune pathogenesis network. The five bioactive compounds detailed in this review show a range of immunomodulatory activities. They impact significant pathways such as NF-κB, MAPK, PI3K/AKT/mTOR, JAK-STAT, PKC, and Nrf2, regulating oxidative stress and inflammatory mediators. These bioactive compounds are shown as coloured circles and are indicated alongside their respective molecular targets (Blue: Apigenin, Yellow: Quercetin, Orange: Oleanolic acid, Green: Betulinic acid, Grey: β-Sitosterol). The bold red arrows indicate the downregulation of respective pathways, and bold green arrows indicate the upregulation of the particular pathway. The entities of bold square boxes represent different signaling molecules/complexes. The entities of dotted square boxes represent different receptors for signaling pathways. The entities of circle boxes represent the inducers/activators of the particular pathways. The entities in star-shaped boxes represent different stimulus/inducers for signaling pathways. → represents direct activation and ⊣ represents inhibition.

Neuroinflammation is an associated factor in numerous neurodegenerative diseases such as Alzheimer’s disease (AD), Parkinson’s disease (PD), Huntington’s disease (HD), and multiple sclerosis (MS). These neurodegenerative diseases result in the progressive and irreversible loss of neurons in the brain due to various stimuli such as radiation, injury, stress, or infection. Prolonged activation of the central nervous system (CNS)-resident macrophage-like immune cells and overproduction of pro-inflammatory cytokines result in chronic CNS neuroinflammation [42]. Major molecular pathways are explained in previous sections, and the PKC pathway is involved in the progression of neuroinflammation, leading to autoimmune diseases. Figure 6 shows the comprehensive immunomodulatory effect of the five bioactive compounds on various signaling pathways. Multiple studies have shown that the bioactive compounds have neuroprotective effects affecting the inflammatory pathways linked to autoimmune pathogenesis.

### 5.1. PKC Pathway

PKC is a family of serine-threonine kinases. They control several cellular responses, such as gene expression, cell proliferation, protein secretion, and inflammatory response. ROS triggers PKC through redox signaling, where the oxidation of their cysteine residues activates PKC. The effect of bioactive compounds such as QU on ROS and PKC activity could be mediated by antioxidants [31]. The neuroprotective effects of BS are correlated with reduced levels of oxidative stress. The antioxidant effect of BS was attributed to their ability to donate electrons from their hydroxyl or carboxyl groups directly to the free radical, thus neutralizing it [43]. Various studies proved that inflammation, initiated by ROS and induced by NF-κB, was an important event in neurotoxicity. Circulating pro-inflammatory cytokines IFN-γ, IL-1β, and TNF-α, were associated with activating extrinsic apoptotic pathways, and apoptosis was observed in neurons. The API effect in neuroinflammation inhibits oxidative stress and regulates mitochondria-mediated neuron apoptosis. API inhibited neuroinflammation via downregulating the TLR4/NF-κB signaling pathway and therefore suppressed neuronal apoptosis expressed as inhibiting mitochondria-mediated apoptosis pathway [20]. API has been reported to exercise its neuroprotective effect by reducing iNOS and nitric oxide NO expression in microglial cells and macrophages [5].

Various neuroinflammatory diseases get triggered due to the immunomodulation of different inflammatory pathways. Bioactive compounds have shown significant effects on these pathways, which proved helpful in curing diseases. Some of them are discussed as follows:

### 5.2. Alzheimer Disease (AD)

AD is a chronic neurodegenerative disease that usually starts slowly and deteriorates over time with increasing age. Bioactive compounds such as QU have an anti-Alzheimer activity that witnesses various mechanisms of action, both direct and indirect, at a cellular and molecular level. QU bioavailability is enhanced as its use as a CNS drug that can cross the blood-brain barrier (BBB). Being an important compound as anti-AD, structural manipulations on QU can provide a drug candidate that limits the progression of the disease [31]. Several mechanisms were recommended for the neuroprotective actions of QU, including inhibition of processes such as Aß aggregation, NFTs formation, amyloid precursor protein (APP) cleaving enzyme (BACE1), acetylcholinesterase (AChE), and others reducing the oxidative stress in AD. QU is known to inhibit the fibrils aggregation of Aß and interrupts the maturation of fibrils by forming hydrophobic interactions and hydrogen bonds with the ß-sheet structure of Aß. QU and other bioactive molecules are helpful to improve neurogenesis and neural longevity by controlling signaling pathways such as P13K, AKT/PKB tyrosine kinase, and PKC in AD.

Oxidative stress induced tau protein phosphorylation is one of the main downstream targets of the PI3K/AKT pathway, which QU blocks. This recommends that QU’s neuroprotective effects are employed by tau anti-hyperphosphorylation, mostly in neurodegenerative disorders such as AD and Parkinson’s disease. Similarly, various other mechanisms of action (ERK1/2, Nrf2, PI3K/AKT, JNK, MAPK pathways) for QU are its antioxidant potential, which directly suppresses AD. Other compounds such as OA treatment significantly increased the expression levels of brain-derived neurotrophic factor (BDNF), CaMKII, cAMP response element-binding (CREB), PKC, and TRKB in an AD model. OA significantly hinders the Aß induced ß23-35 differentiation of NSCs into astrocytes by down-regulating the JAK/STAT signaling pathway through increasing NGN1 expression. These outcomes suggest that OA might impede the progress of AD [24].

Apart from the usual immunoregulatory effects of API, BA, and BS, it is observed in various studies that QU and OA play a major role in downregulating neuroinflammatory mediators, effector immune cells, oxidative stress, and upregulating regulatory immune cells and antioxidants, thus act as antioxidant, neuroprotective agents against AD.

### 5.3. Multiple Sclerosis (MS)

MS is an autoimmune disease mediated by an immunogenic response of auto-inflammatory T cells against the myelin sheath protecting the neurons. Dysregulation of dendritic cells (DCs) function in MS can result from several possible reasons but are not limited to T-cell anergy in response to determining antigens displayed by long-lived lymphoid DCs and functional aberration of DCs.

Bioactive compounds such as API prevent neuronal apoptosis by protecting the neurons against inflammatory stresses. API’s potential in modulating neuroinflammation and its subsequent effects on immune cell activity was shown by suppressing iNOS and NO expression in microglial cells and macrophages. API can cross the BBB and has been shown to apply anti-inflammatory effects on primary microglial cells by inhibiting p38 and JNK [5]. The neuroprotective effects of API in experimental autoimmune encephalomyelitis (EAE) are due to its inhibition of DCs’ phenotypic and functional maturation and its resulting polarization of CD4 T helper cells. The effects of API on human peripheral blood DCs suggested that API reduced cell surface expression of key antigen presentation and co-stimulatory markers and reduced the production of pro-inflammatory cytokines in LPS-matured DCs treated with API in a RelB-dependent manner. NF-κB protein RelB is a master regulator of DC differentiation and is suggested as a possible target for manipulating DC differentiation, especially in autoimmune diseases. In mature DCs, RelB is upregulated and directed into the nuclei in response to several maturation signals. The API-induced blood DCs leads to T-cell polarisation from Th1 and Th17 cells towards Treg cells [6,44].

Thus, targeting an oxidative stress mediator and a DC-specific anti-inflammatory agent could be crucial in resolving CNS inflammation and the subsequent pathologies in various neurodegenerative diseases. Additionally, because of their moderately long half-life, delayed plasma clearance, and deliberate metabolism in the liver, API, QU, BA, BS, and OA have considerable potential to be developed as a safer, more cost-effective treatment for neurodegenerative diseases affecting the major nodal points of various stimulatory pathways.

## 6. Other Immune Responses

### Neutropenia

Neutropenia is a condition that is considered to have a decrease in absolute neutrophil counts (ANC). It is a common adverse event associated with many cytotoxic chemotherapy drugs. Chemotherapeutic-induced neutropenia is a potentially serious and life-threatening event that may occur secondary to therapy with various drugs. Neutropenia may be immune-mediated or due to direct inhibition of the bone marrow precursor cells. It either results from a failure to produce neutrophils in the bone marrow or from peripheral destruction [41].

In case of infection under neutropenic conditions, γδ T cells can play an important role in recruiting neutrophils to sites of bacterial infection. γδ T cells are found to govern neutrophil-mediated host defence against *Streptococcus pneumoniae* lung infection by promoting TNF-α and CXCL2 in the infected tissue. They are the main producers of IL-17, consequently stimulating the recruitment of neutrophils. IL-17-producing γδ T cells have been shown to control the neutrophil influx under infection. QU is known to increase γδ T cell proliferation and Tregs levels [44]. Tregs can encourage neutrophil recruitment through the production of CXCL8. It is known that neutrophils express death receptors and are susceptible to TNF-triggered apoptosis. As QU is also shown to reduce TNF-α activity, this can help minimize TNF-triggered apoptosis [45]. Additionally, the broad-spectrum immunotherapeutic effects of these major bioactive compounds can also help fight infection during the neutropenic condition by restoring balance in the immune environment by regulating signaling pathways and immune cells.

There are various other immune response mechanisms and molecular pathways that get perturbed due to different intrinsic or extrinsic factors, and the five natural bioactive compounds API, QU, BA, OA, and BS have been studied in detail to have targeted those pathways individually and have immunomodulatory effects in the diseased microenvironment. Some of these diseased conditions, along with the bioactive compound effects, are listed in Table 2. A combinatorial therapy or concoction of these bioactive compounds can modulate the immune molecular pathways and be useful as natural therapeutics.

## 7. Conclusions

The present review illustrates the impact of plant-bioactive compounds in both healthy and disease microenvironments. The five plant-based compounds, namely API, QU, BA, OA, and BS, are safe and are used as dietary supplements for their broad range of biological effects in individuals. Several medicinal herbs are known to contain these bioactive compounds in varying proportions. Various herbal supplements positioned for immunomodulatory properties such as Immusante (a herbal formulation developed and recommended as immunotherapeutic support in conditions where immunity is compromised) contain at least four bioactive constituents, namely, API, QU BA, and OA. However, products such as Immusante may contain additional phytoconstituents, which may act synergistically. Therefore, the current review emphasizes the combined physiological effects of these bioactive compounds, including immunomodulatory, anti-inflammatory, antioxidant, anti-mutagenic, anti-microbial, anti-viral, anti-tumour, anti-allergic, neuroprotective, and cardioprotective effects.

The present review includes various static signaling networks detailing all the inflammation, redox metabolism, proliferation, angiogenesis, and cell survival-related effects of these bioactive components. They mediate immunomodulation by suppressing key elements of NF-κB, MAPK, PI3K-AKT-mTOR, JAK-STAT, and PKC pathway signaling axis, which reduces pro-inflammatory cytokines such as TNF-α, IL-1β, IL-6, and IL-8, enzymes such as COX and iNOS, and factors such as ROS, STAT3, VEGF, and PDL1. A combination of API, QU, OA, BA, and BS can modulate respective pathways to reduce the inflammatory mediators. All five compounds enhance antioxidant function and cell death by upregulating the Nrf2 pathway, apoptosis, and cell cycle arrest. Notably, phytochemicals have great potential to activate cell cycle checkpoints and induce cell cycle arrest in cancer cells to limit their growth. A concoction of API, QU, OA, BA, and BS can be used as anti-cancer therapeutic agents by impacting the cell cycle arrest through various mechanisms. Thus, a combination of these elements can prove to be beneficial and immunomodulatory against multiple molecular targets.

The myriad of effects exhibited by selected bioactive compounds may act synergistically to boost the protective effect of the immune system, thus contributing to enhanced therapeutic effects. From a future perspective, the ability to sensitively tune fundamental mechanisms in inflammation and cell survival renders these compounds particularly useful in being adopted as prodrugs and therapeutic molecules against various disorders. We recommend further research to reveal the most effective bioactive compounds and their combinations in attenuating associated inflammatory dysregulations, neurodegenerative, autoimmune disorders, cancer, and other diseases.

## Figures and Tables

**Table 1:** Selected natural bioactive compounds, their molecular targets, and biological functions.

Compounds	Class	Source	Molecular Targets	Biological Function	References
Apigenin (API)	Flavone	Fruit skins, red pepper, and tomato skin	AKT, ERK, caspase 12, caspase-3, MAPK, ROS, COX-2, IL-6, TNF-α, IL-1, iNOS	Anti-inflammatory, anti-carcinogenic, neuroprotective	[7,8,10]
Quercetin (QU)	Flavonol	Onion, red wine, olive oil, berries, and grapefruit	PKC, AP-1, H_2_O_2_, iNOS, MMP-9, MMP-2, COX-2, ERK	Antioxidant, anti-inflammatory	[9,10,17]
Betulinic acid (BA)	Triterpenoids	The bark of white birch, ber tree, sycamore	Bax, Bcl-2, Bcl-xL, caspase-3,-8, and -9, CD95, cyclin D1, JNK, NF-κB, p53, VEGF	Neuroprotective, reduce risk of vascular disease	[12,13,18]
Oleanolic acid (OA)	Triterpenoids	Olives, bilberries, persimmon, Jujube	Caspase-3 and -8, mTOR, NF-κB, VEGF	Anti-tumour, anti-HIV, hepatoprotective activities, diuretic, antimycotic, anti-inflammatory	[14,19]
β-Sitosterol (BS)	Phytosterol	Soy products, vegetable oil, peanuts, flaxseed	NF-κB, Nrf2, Trx, ROS	Antioxidant, antidiabetic, microbicide, anti-inflammatory, hypolipidemic, anti-atherosclerotic	[15,16,20]

**Table 2:** Immune responses of natural bioactive compounds.

Compounds	Inflammation/Oxidative Stress/ Autoimmune Diseases/Cancer		Others
Apigenin (API)	NF-κB, MAPK(ERK-p38-JNK), JAK/STAT, AKT	↓	Obesity, diabetes, cardiac injury, IBD [13], viral infection [5]
Quercetin (QU)	NF-κB, MAPK(ERK-p38-JNK), PI3K, AKT, ROS	↓	Cardiovascular disease and hypertension, diabetes, IBD, viral infection [5], Bacterial infection [46], asthma allergic diseases [47]
	Nrf2	↑	
Oleanolic acid (OA)	MAPK-ERK, NF-κB (IKK-α/β), PI3K/AKT, STAT, ROS	↓	Diabetes [10], osteoporosis, insulin metabolism, hepatic disease [24]
	Nrf2	↑	
Betulinic acid (BA)	IKK-α/β, ROS, MAPK (ERK-p38-JNK)	↓	Glucose metabolism, lipid regulation, diabetes, viral infection, obesity, spinal cord injury [48], cardiac disease [17]
	Nrf2	↑	
β-Sitosterol (BS)	NF-κB, ROS	↓	IBD, diabetes [12]
	Nrf2	↑	
